# Ameliorative effects of polyunsaturated fatty acids against palmitic acid-induced insulin resistance in L6 skeletal muscle cells

**DOI:** 10.1186/1476-511X-11-36

**Published:** 2012-03-12

**Authors:** Keisuke Sawada, Kyuichi Kawabata, Takatoshi Yamashita, Kengo Kawasaki, Norio Yamamoto, Hitoshi Ashida

**Affiliations:** 1 Department of Agrobioscience, Graduate School of Agricultural Science, Kobe University, Kobe, Hyogo 657-8501, Japan; 2 J-Oil Mills Inc., Yokohama, Kanagawa 230-0053, Japan; 3 Food Science Research Center, House Wellness Foods Corporation, Itami, Hyogo 664-0011, Japan; 4 Department of Bioscience, Fukui Prefectural University, Fukui 910-1195, Japan

**Keywords:** Insulin resistance, Glucose uptake, L6 skeletal muscle cells, Palmitic acid, Arachidonic acid, Eicosapentaenoic acid

## Abstract

**Background:**

Fatty acid-induced insulin resistance and impaired glucose uptake activity in muscle cells are fundamental events in the development of type 2 diabetes and hyperglycemia. There is an increasing demand for compounds including drugs and functional foods that can prevent myocellular insulin resistance.

**Methods:**

In this study, we established a high-throughput assay to screen for compounds that can improve myocellular insulin resistance, which was based on a previously reported non-radioisotope 2-deoxyglucose (2DG) uptake assay. Insulin-resistant muscle cells were prepared by treating rat L6 skeletal muscle cells with 750 μM palmitic acid for 14 h. Using the established assay, the impacts of several fatty acids on myocellular insulin resistance were determined.

**Results:**

In normal L6 cells, treatment with saturated palmitic or stearic acid alone decreased 2DG uptake, whereas unsaturated fatty acids did not. Moreover, co-treatment with oleic acid canceled the palmitic acid-induced decrease in 2DG uptake activity. Using the developed assay with palmitic acid-induced insulin-resistant L6 cells, we determined the effects of other unsaturated fatty acids. We found that arachidonic, eicosapentaenoic and docosahexaenoic acids improved palmitic acid-decreased 2DG uptake at lower concentrations than the other unsaturated fatty acids, including oleic acid, as 10 μM arachidonic acid showed similar effects to 750 μM oleic acid.

**Conclusions:**

We have found that polyunsaturated fatty acids, in particular arachidonic and eicosapentaenoic acids prevent palmitic acid-induced myocellular insulin resistance.

## Background

Insulin resistance is an impaired response to insulin in specific organs or cells such as liver, fat and muscle, and is strongly associated with the development of obesity and type 2 diabetes [1]. Elevated plasma free fatty acid levels is an important factor, because it causes insulin resistance in skeletal muscle, the major site for blood glucose disposal [2]. Thus, many studies have been reported on the relationship between fatty acids and insulin resistance, and revealed that saturated fatty acids, particularly palmitic acid, induce insulin resistance in myotubes [3], whereas unsaturated fatty acids do not [4,5]. In skeletal muscle, insulin resistance is mediated by the intramyocellular accumulation of the metabolites of saturated palmitic acid, namely diacylglycerol (DAG) and ceramide. DAG downregulates insulin-sensitive glucose transporter type 4 (GLUT4) and insulin receptor (IR) by activating the inflammatory transcription factor nuclear factor (NF)-κB [6]. Ceramide inhibits protein kinase B (PKB/Akt) activity which plays an important role in insulin signaling [7,8]. The levels of these metabolites progressively increase as insulin resistance worsens [9-11], and further decrease glucose uptake activity in myotubes.

There is an increasing demand for drugs and functional foods that are capable of regulating blood glucose levels. Several unsaturated fatty acids, including palmitoleic and oleic acids, were reported to ameliorate palmitic acid-induced insulin resistance in myotubes [4,12,13]. Coll *et al. *(2008) reported that oleic acid inhibited intramyocellular DAG accumulation by enhancing β-oxidation of palmitoyl CoA and upregulating diacylglycerol acyltransferase 2, an enzyme that synthesizes triacylglycerol from DAG, which ultimately inhibited palmitic acid-induced downregulation of IR [12]. Chen *et al. *have reported that berberine, an isoquinoline alkaloid, improves palmitic acid-induced insulin resistance in L6 myotubes by inhibiting peroxisome proliferator-activated receptor (PPAR)-γ [14]. Other than these compounds, little is known about inhibitors of palmitic acid-induced insulin resistance in muscle cells.

In these earlier studies, insulin resistance was evaluated based on downregulation of IR and GLUT4 expression or decreased radioisotope-labeled glucose uptake [15-22]. We recently reported an enzymatic 2DG uptake assay, which had greater processing capacity compared with these conventional tools [23,24]. This enzymatic assay enables us to measure 2DG uptake into myotubes cultured in a 96-well microplate by measuring the fluorescence of resorufin, which is derived from resazurin, and is appropriate to screen for compounds capable of regulating 2DG uptake, including polyphenols [25,26]. In this study, we report our development of a high-throughput procedure to screen for compounds that can prevent palmitic acid-induced myocellular insulin resistance using this enzymatic 2DG uptake assay, and assessment of the anti-insulin-resistant effects of unsaturated fatty acids.

## Results

### Determination of optimum treatment time and concentration of palmitic acid

**Figure 1:**
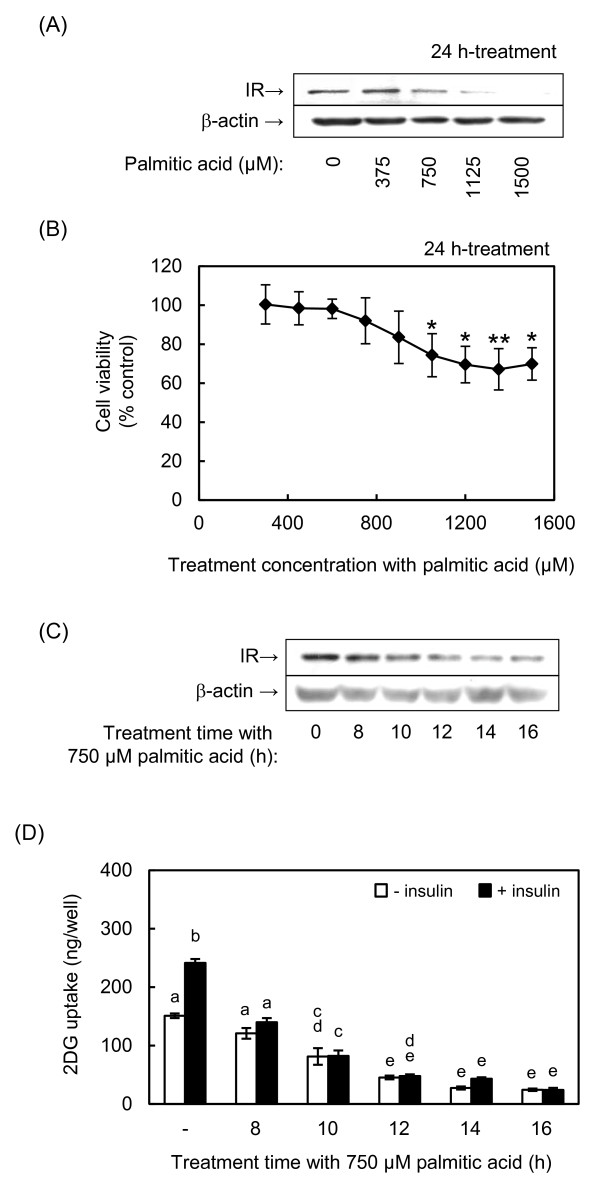
**Palmitic acid downregulates IR expression and 2DG uptake activity**. Differentiated L6 cells were incubated with the indicated concentrations of palmitic acid for the indicated times. Dose-dependent effect of palmitic acid on **(A) **IR expression detected by western blotting, and **(B) **cell viability determined by crystal violet staining. **(C) **Time-dependent effect of palmitic acid on IR expression determined by western blotting. **(D) **Time-dependent effects of palmitic acid on 2DG uptake activity in the absence or presence of 100 nM insulin. * and ** indicate significant difference vs control at *p *< 0.05 and *p *< 0.01, respectively, for panel (B). Different letters indicate significant changes (*p *< 0.05) for panel (D).

To induce insulin resistance in muscle cells, we treated differentiated L6 skeletal muscle cells with palmitic acid according to a method reported by Chaves *et al. *[7]. Treatment-time and concentration of palmitic acid were defined by evaluating the decreases in IR expression, cellviability and 2DG uptake activity. Treating cells with 750 μM palmitic acid for 24 h significantly decreased IR expression (Figure 1A). After we confirmed that palmitic acid did not show significant cytotoxicity by 900 μM, the treatment concentration was fixed at 750 μM according to the previous reports [4,6] (Figure 1B). This concentration of palmitic acid also decreased IR expression after 14 and 16 h of treatment (Figure 1B), and decreased basal and insulin-induced 2DG uptake activity in a time-dependent manner (Figure 1C). The decrease in 2DG uptake activity was well correlated with the decrease in IR expression. Based on these results, we prepared insulin-resistant cells in the following experiments by treating differentiated L6 cells with 750 μM palmitic acid for 14 h.

### Effect of fatty acids on myocellular glucose uptake activity

**Figure 2:**
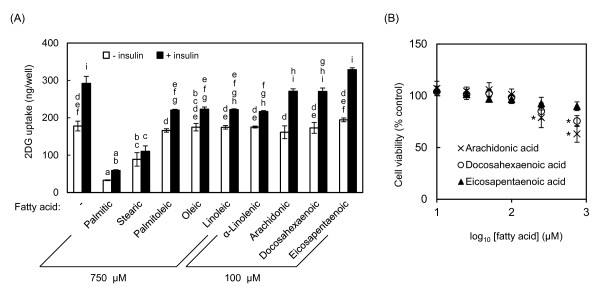
**Fatty acid-induced decreases in 2DG uptake activity and viability of L6 cells**. **(A) **Differentiated L6 cells were incubated with the indicated fatty acid (750 μM palmitic, stearic, palmitoleic, oleic, linoleic or α-linolenic acids; or 100 μM arachidonic, docosahexaenoic or eicosapentaenoic acid) for 14 h. 2DG uptake activity was measured in the absence or presence of 100 nM insulin. **(B) **Cell viability was determined by crystal violet staining after treatment with the indicated concentrations of arachidonic, docosahexaenoic and eicosapentaenoic acids for 24 h. Different letters indicate significant changes (*p *< 0.05) for panel (A). * indicates significant difference vs control at *p *< 0.05 for panel (B)

We used the enzymatic 2DG uptake assay to evaluate the effects of several saturated and unsaturated fatty acids commonly contained in food on the development of insulin resistance. Palmtic acid (16:0) significantly decreased glucose uptake activity to 19% and 33% in the absence and presence of insulin, respectively (Figure 2A). Stearic acid (18:0) also significantly decreased 2DG uptake, although its effect was weaker than that of palmitic acid.

On the other hand, the unsaturated forms of these fatty acids, such as palmitoleic (16:1), oleic (18:1), linoleic (18:2) and α-linolenic acid (18:3), only affected 2DG uptake activity in the presence of insulin. Other polyunsaturated fatty acids, including arachidonic (20:4), docosahexaenoic (22:6) and eicosapentaenoic acids (20:5), were tested at a lower concentration (100 μM) because arachidonic and docosahexaenoic acids were cytotoxic at 750 μM (Figure 2B). These three polyunsaturated fatty acids did not affect 2DG uptake activity (Figure 2A). Palmitic acid showed the strongest effect in terms of decreasing 2DG uptake activity. These results confirm that palmitic acid is an appropriate inducer of myocellular insulin resistance.

### Effects of unsaturated fatty acids on palmitic acid-induced insulin resistance

**Figure 3:**
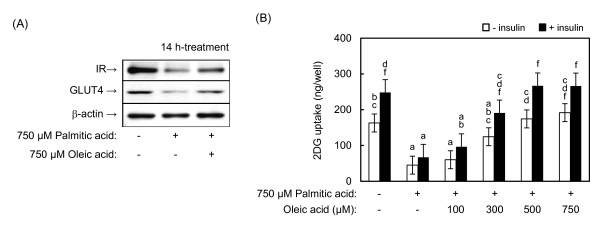
**Effects of oleic acid on palmitic acid-induced decreases in IR and GLUT4 expression and 2DG uptake activity**. Differentiated L6 myotubes were treated with 750 μM palmitic acid without or with the indicated concentrations of oleic acid for 14 h. **(A) **IR and GLUT4 expression determined by western blotting. **(B) **Dose-dependent effects of oleic acid on 2DG uptake activity in the presence of 750 μM palmitic acid in the absence or presence of 100 nM insulin. Different letters indicate significant changes (*p *< 0.05).

To confirm the effects of oleic acid on palmitic acid-induced insulin resistance, L6 myotubes were simultaneously treated with palmitic acid and oleic acid. In this assay, oleic acid canceled palmitic acid-induced decreases in IR and GLUT4 expression (Figure 3A), as expected. Furthermore, oleic acid dose-dependently inhibited the palmitic acid-induced decrease in 2DG uptake activity (Figure 3B). These results indicate that the enzymatic 2DG uptake assay using palmitic acid-treated cells is useful to screen for compounds that regulate insulin resistance in skeletal muscle cells.

**Figure 4:**
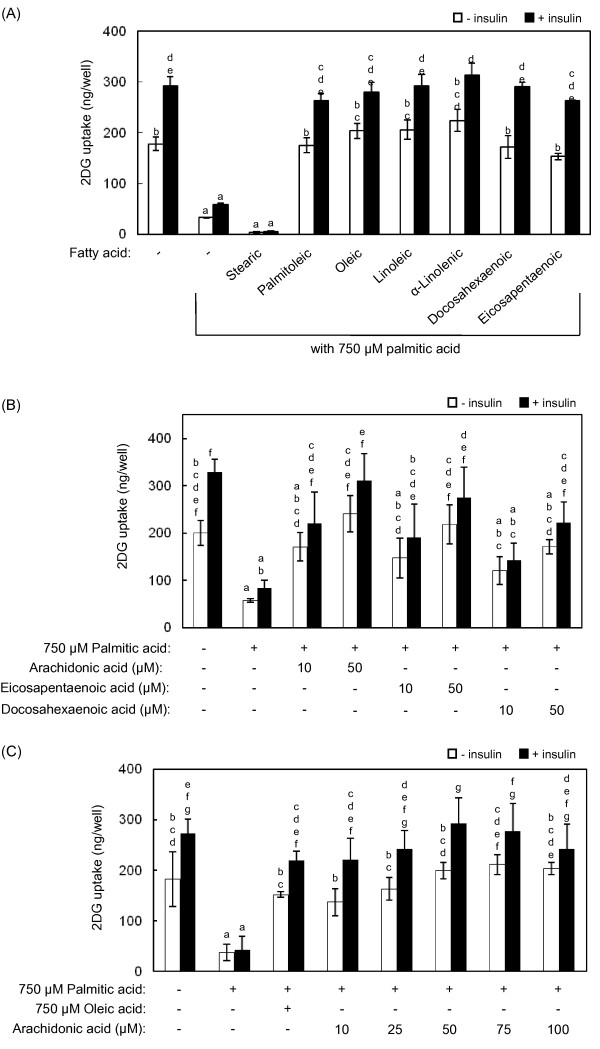
**Effects of arachidonic acid and other fatty acids on palmitic acid-induced decreases in 2DG uptake activity**. Differentiated L6 myotubes were treated with 750 μM palmitic acid without or with the indicated fatty acids [**(A) **750 μM stearic, palmitoleic, oleic, linoleic or α-linolenic acid; or 100 μM docosahexaenoic and eicosapentaenoic acid; **(B) **10 and 50 μM arachidonic, eicosapentaenoic, or docosahexaenoic acid)] for 14 h. **(C) **Differentiated L6 myotubes were treated with 10-100 μM arachidonic acid as in (A). After treatment with the fatty acids, 2DG uptake activity was measured in the absence or presence of 100 nM insulin. Different letters indicate significant changes (*p *< 0.05).

Since 750 μM oleic acid showed the strongest effect on the recovery of 2DG uptake in palmitic acid-treated L6 cells, we evaluated the effects of other fatty acids at 750 μM on palmitic acid-induced insulin resistance. Compared with palmitic acid treatment alone, cotreatment with stearic acid evoked a further decrease in 2DG uptake activity in L6 myotubes (Figure 4A). All of the unsaturated fatty acids including oleic acid could cancel decrease in 2DG uptake activity induced by palmitic acid. Interestingly, polyunsaturated fatty acids were much more effective. For example, 100 μM docosahexaenoic acid and eicosapentaenoic acid elicited similar effects to 750 μM oleic acid (Figure 4A). Moreover, arachidonic acid was more effective than the other polyunsaturated fatty acids at 10 and 50 μM (Figure 4B), with significant effects at 10 μM. It was confirmed that eicosapentaenoic acid was also effective at the lower concentrations. As shown in Figure 4C, significant recovery effect of arachidonic acid on 2DG uptake was observed at 10 μM and reached maximum levels at 50 μM and 75 μM in the presence and absence of insulin, respectively.

**Figure 5:**
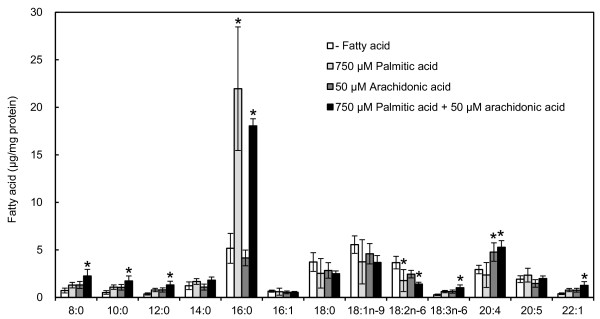
**Effect of exogenously added palmitic and oleic acids on cellular fatty acid contents**. Differentiated L6 myotubes were treated with 750 μM palmitic acid and/or 50 μM arachidonic acid for 14 h. Intracellular fatty acid contents were measured by using a gas chromatography. * indicates significant difference vs control at *p *< 0.05.

We further investigated whether treated fatty acids would incorporated into the cells by measuring fatty acid contents in the cells treated with 750 μM palmitic acid or 50 μM arachidonic acid (Figure 5). It was confirmed that intracellular contents of the treated fatty acids were significantly higher than those of non-treated cells; i.e., content of palmitic acid increased 3-fold while that of arachdonic acid 2-fold. Co-treatment with palmitic and arachidonic acids also increased both fatty acids in the cells.

## Discussion

The *in vitro *evaluation of compounds that can affect insulin resistance in myotubes has long been performed by measuring the changes in uptake of radiolabeled glucose analogs and/or in the expression and phosphorylation of components of the insulin signaling pathways [15-22]. However, these conventional approaches are unsuitable for high-throughput screening, because radiolabeled analogs are costly and require specialized equipments, and both approaches have low processing capacity. Our research group previously reported a high-throughput enzymatic 2DG uptake assay system [23,24]. In the present study, we developed a high-throughput assay based on this previously reported assay system to screen for compounds that can ameliorate insulin resistance in L6 myotubes. Using this system, we found that arachidonic, eicosapentaenoic, and docosahexaenoic acids were more effective than oleic acid [12,13] in terms of ameliorating insulin resistance.

In this study, we first confirmed that saturated palmitic and stearic acids decreased glucose uptake by L6 myotubes, whereas their unsaturated forms (i.e., oleic, palmitoleic, linoleic and α-linolenic acids) did not. These results are consistent with previous reports [4,5]. Indeed, it was reported that myocellular insulin resistance is mediated by the accumulation of the saturated fatty acid metabolites DAG and ceramide. DAG activates novel PKCs that downregulate GLUT4 and IR expression by activating NF-κB [6]. Ceramide inhibits PKB/Akt activity, which plays an important role in insulin signaling [7,8]. Palmitic acid is a stronger inducer of the accumulation of these metabolites and the transcriptional activity of NF-κB compared as stearic acid [3], which is correlated with the greater decrease in glucose uptake activity in palmitic acid-treated cells. These facts and the obtained results indicate that our assay with palmitic acid-induced insulin resistant L6 myotubes is suitable to screen for compounds that can induce or prevent insulin resistance.

Using palmitic acid as an inducer of insulin resistance, we tested the anti-insulin resistant effects of several fatty acids. All of the unsaturated fatty acids tested in this study, namely palmitoleic, oleic, linoleic, linolenic, arachidonic, docosahexaenoic and eicosapentaenoic acids, ameliorated palmitic acid-induced decrease in 2DG uptake (Figure 4). Unsaturated fatty acids, in particular polyunsaturated fatty acids, are known to be oxidized easily. Therefore, to investigate whether the recovery of 2DG uptake was dependent on the exogenously added fatty acids per se or their oxidized derivatives, we conducted an experiment with an antioxidant, butylated hydroxytoluene (BHT). In the presence of 0.2 mM BHT in the medium, palmitic acid-induced decrease and its amelioration by arachidonic acid were confirmed(data not shown). However, 2DG uptake was decreased by treatment with BHT alone, indicating that BHT itself affected cellular functions under our experimental conditions. Meanwhile, Figure 5 shows that treated palmitic and arachidonic acid were increased in the cells. These results suggest that the exogenously added arachidonic acid and other unsaturated fatty acids are incorporated into the cells, and contribute to their anti-insulin resistant effects.

Of the tested unsaturated fatty acids, oleic acid has been a focus of many researches. The effects of oleic acid were reported to be dependent on its ligand activity for PPARα which plays essential roles in regulation of lipid metabolism [27]. PPARα activation improves intramyocellular DAG accumulation by accelerating β-oxidation and upregulating DAG acyltransferase 2, an enzyme that synthesizes triacylglycerol from DAG [12]. The effects of other fatty acids would depend, at least in part, on PPARα because of its high affinity for unsaturated fatty acids [27,28]. Our results indicate that the effects of arachidonic, eicosapentaenoic and docosahexaenoic acids at much lower concentrations compared with oleic acid (Figure 4A and 4C). This result strongly suggests that bioactive metabolites of these polyunsaturated fatty acids are involved in underlying mechanism. Indeed, prostaglandin E2, a metabolite of n-6 polyunsaturated fatty acids, ameliorated the palmitic acid-induced inflammation, and recovered PKB/Akt phosphorylation level and glucose uptake in muscle cells [29]. Therefore, the results obtained for polyunsaturated fatty acids in our assay are mainly due to their PPARα ligand activity or anti-inflammatory activity. However, the mechanisms by which arachidonic, eicosapentaenoic and docosahexaenoic acids ameliorated insulin resistance, which seem to be independent of the mechanisms exploited by the other unsaturated fatty acids tested, remain unclear. Further studies are needed to clarify the underlying mechanisms.

## Conclusions

We developed a high-throughput assay to screen for compounds that can improve palmitic acid-induced insulin resistance by modifying our previously established enzymatic 2DG uptake assay [23,24]. Using the developed assay, we determined the ameliorative effects of several polyunsaturated fatty acids on insulin resistance. Notably, arachidonic, eicosapentaenoic and docosahexaenoic acids showed were more potent than the other unsaturated fatty acids tested in this study.

## Methods

### Materials

Minimum essential medium (MEM), 2-deoxyglucose (2DG), glucose-6-phosphate dehydrogenase (G6PDH), resazurin and stearic acid (Sigma, St. Louis, MO); diaphorase and β-nicotinamide adenine dinucleotide phosphate (β-NADP+; Oriental Yeast, Tokyo, Japan); arachidonic acid, docosahexaenoic acid and eicosapentaenoic acid (Cayman Chemical,Ann Arbor, MI); fetal bovine serum (FBS; Biological Industries, Kibbutz Beit Haemek, Israel); palmitoleic acid and bovine serum albumin (BSA; Nakarai Tesque, Kyoto, Japan) and Lumi-Light Plus Western Blotting Substrate^® ^(Roche Diagnostics, Mannheim, Germany) were purchased from commercial sources. Anti-GLUT4, anti-IR, anti-goat IgG and anti-rabbit IgG antibodies were purchased from Santa Cruz Biotechnology (Santa Cruz, CA). All other reagents were purchased from WAKO Pure Chemicals (Osaka, Japan), unless otherwise specified.

### Cell culture

Rat skeletal muscle L6 cells (passages 27-38) were maintained in MEM supplemented with 10% FBS, 100 U/mL penicillin, and 100 μg/mL streptomycin at 37°C in a humidified atmosphere with 5% CO2. To induce differentiation into myotubes, L6 cells were seeded on 96-well plates (4 × 103 cells/0.2 mL), 60-mm dishes (1.2 × 105 cells/4 mL), or 35-mm dishes (4 × 104 cells/1 mL) in culture medium. After 2 days, the medium was replaced with MEM containing 2% FBS and antibiotics, which was changed every other day. L6 cells were used for experiments after 5 days of differentiation. Fatty acid-containing media were prepared by conjugating fatty acids to BSA, as previously described [7], but with some modifications. Briefly, palmitate was dissolved in ethanol and diluted in MEM containing 2% (*w/v*) BSA. The cells were incubated for 14 h in MEM containing 2% FBS and 2% BSA in either the presence or absence of fatty acids. The treated cells were used for western blotting analysis and 2DG uptake assays.

### Western blotting

After treatment with fatty acids, the cells plated on 60-mm dishes were washed twice with Krebs-Ringer HEPES buffer (KRH; 50 mM HEPES, pH 7.4, 137 mM sodium chloride, 4.8 mM potassium chloride, 1.85 mM calcium chloride, 1.3 mM magnesium sulfate) and homogenized with RIPA buffer [50 mM Tris, pH8.0, 150 mM sodium chloride, 0.5% (*w/v*) sodium deoxycholate, 1% (*w/v*) sodium dodecyl sulfate, 1% (*v/v*) Nonidet P-40] containing protease and phosphatase inhibitors. The cell lysate was incubated on ice for 1 h with occasional mixing. After centrifugation at 16,000 × g for 20 min at 4°C, the supernatant was collected and used as whole protein. Aliquots of whole protein were separated on 10% polyacrylamide gels and transferred to polyvinylidene difluoride membranes (Biotrace, Pall Corporation, Port Washington, NY). After blocking with Blocking one^® ^(Nakarai Tesque), the membranes were treated with appropriate specific primary antibodies for 1 h at room temperature, followed by the corresponding horseradish peroxidase-conjugated secondary antibody for 1 h at room temperature. Specific immune complexes were detected using ECL plus kits (GE Healthcare Bio-Sciences Corporation, Tokyo, Japan).

### Glucose uptake assay

Glucose uptake in L6 myotubes was measured using an enzymatic 2DG uptake assay as previously described [23,24], with some modifications. L6 myotubes on a 96-well plate were serum-starved in 0.2% BSA/MEM for 18 h and were treated with DMSO and insulin (final concentration, 0.1 μM) in the same media. The cells were washed twice with KRH buffer containing 0.1% (*w/v*) BSA and incubated with 1 mM 2DG in 0.1% (*w/v*) BSA/KRH buffer for 20 min at 37°C in 5% CO2. They were then washed twice with 0.1% (*w/v*) BSA/KRH buffer, lysed with 0.1 N sodium hydroxide, warmed at 60°C for 10 min, and dried at 85°C for 50 min. The dried cell lysate was solubilized with 0.1 N hydrochloric acid and 200 mM triethanolamine (TEA) (pH 8.1), and gently stirred using a microplate shaker. The lysate was mixed with an assay cocktail [50 mM TEA, pH 8.1, 50 mM KCl, 0.02% (*w/v*) BSA, 0.1 mM β-NADP+, 2 units diaphorase, 150 units G6PDH, 2 μM resazurin] on another 96-well plate and incubated at 37°C for 50 min. The fluorescence of resorufin was measured at 570 nm with excitation at 530 nm using a Wallac 1420 ARVOsx (Perkin-Elmer, Boston, MA). The 2DG concentration in each well was calculated based on a standard curve generated with a 2DG-6-phosphate solution.

### Cytotoxicity

The cytotoxicity of fatty acids was determined by crystal violet staining [25]. Differentiated L6 cells on 96-well microplate were treated with ethanol or fatty acids (300, 450, 600, 750, 900, 1050, 1200, 1350 and 1500 μM for Figure 1B; and 10, 25, 50, 100, 250 and 750 μM for Figure 2B) in MEM containing 2% BSA for 24 h, and then stained with 2% ethanol containing 0.2% (w/v) crystal violet for 10 min. The wells were washed three times with tap water, and the stained cells were extracted with 50% ethanol containing 0.5% (w/v) sodium dodecyl sulfate. The absorbance at 570 nm with a reference wavelength of 630 nm was measured using the Wallac 1420 ARVOsx.

### Measurement of fatty acid contents

L6 myotubes on a 35-mm culture dish were incubated for 14 h in MEM containing 2% FBS and 2% BSA in either the presence or absence of palmitic and arachidonic acids. The treated cells were washed with 2% BSA/KRH for 3 times, and with phosphatydyl buffer serine twice, and collected with phosphate buffered saline (137 mM sodium chloride, 8.10 mM sodium phosphate dibasic, 2.68 mM potassium chloride, 1.47 mM potassium phosphate monobasic). Intracellular fatty acid contents were measured by using a gas chromatography according to previous report with slight modification [30]. Briefly, lipids were extracted from the cells by chloroform:methanol (2:1, v/v). hydolized and methylated with potassium hydroxide in methanol, and subjected to a GC2010 gas chromatograph (Shimadzu Co., Kyoto, Japan) equipped with DB23 column (Agilent J&W, Folsom, CA).

### Statistical analysis

Data are shown as means ± SD of results from three independent experiments. Statistical analyses were performed using the Tukey-Kramer test, except for the cytotoxicity tests (Dunnet test, for Figures 1B and 2B). Values of *p *< 0.05 were considered significant.

## Abbreviations

BSA: Bovine serum albumin; BHT: Butylated hydroxytoluene; DAG: Diacylglycerol; 2DG: 2-deoxyglucose; FBS: Fetal bovine serum; GLUT4: Glucose transporter type 4; G6PDH: Glucose-6-phosphate dehydrogenase; IR: Insulin receptor; KRH: Krebs-Ringer HEPES buffer; β-NADP^+^: β-nicotinamide adenine dinucleotide phosphate; NF-κB: Nuclear factor-κB; PKB/Akt: Protein kinase B; PPAR: Peroxisome proliferator-activated receptor.

## Competing interests

The authors declare that they have no competing interests.

## Authors' contributions

KS conceived the study, its design and coordination, performed all experiments and statistical analysis. KS and HA discussed results and made the manuscript. TK measured fatty acid contents. NY and HA participated in the design and coordination of the study and discussion of results. K Kawabata, TY, and K Kawasaki participated in the design and coordination of the study. All authors read and approved the final manuscript.
